# Edmund Townsend

**Published:** 1918-11

**Authors:** 


### OBITUARY

Readers are requested to report promptly the death of all physicians in Western New York, or former residents of this region, or graduates of any medical school in Western New York, and to notify the families of the deceased of our desire to publish adequate Obituary notices.

---

**Edmund Townsend,** Fairport, N. Y.; University of Buffalo, N. Y., 1885; aged 57; recently a surgeon on a steamer in the merchant service; who was operated on at the Primrose Hospital, Batavia, N. Y., in the early spring; died in Washington, D. C., September 4, from carcinoma of the stomach.

